# Gene expression profiling and functional analysis of angiogenic markers in murine collagen-induced arthritis

**DOI:** 10.1186/ar3922

**Published:** 2012-07-20

**Authors:** Yvonne Raatz, Saleh Ibrahim, Marc Feldmann, Ewa M Paleolog

**Affiliations:** 1 Kennedy Institute of Rheumatology, Imperial College London, 65 Aspenlea Road, London W6 8LH, UK; 2 Department of Dermatology, Venerology and Allergology, Medical Faculty of the Leipzig University, Johannisallee 30, 04103 Leipzig, Germany; 3 Department of Dermatology, University of Lübeck, Ratzeburger Allee 160, D-23538 Lübeck, Germany; 4 Kennedy Institute of Rheumatology, University of Oxford, 65 Aspenlea Road, London W6 8LH, UK

## Abstract

**Introduction:**

Dysregulated angiogenesis is implicated in the pathogenesis of rheumatoid arthritis (RA). To provide a more profound understanding of arthritis-associated angiogenesis, we evaluated the expression of angiogenesis-modulating genes at onset, peak and declining phases of collagen-induced arthritis (CIA), a well-established mouse model for RA.

**Methods:**

CIA was induced in DBA/1 mice with type II collagen. Functional capillary density in synovial tissue of knee joints was determined by intravital fluorescence microscopy. To assess the ability of arthritic joint homogenates to induce angiogenesis, an endothelial chemotaxis assay and an *in vivo *matrigel plug assay were employed. The temporal expression profile of angiogenesis-related genes in arthritic paws was analysed by quantitative real-time RT-PCR using an angiogenesis focused array as well as gene specific PCR. Finally, we investigated the therapeutic effect of a monoclonal antibody specifically blocking the binding of VEGF to neuropilin (NRP)-1.

**Results:**

Although arthritic paw homogenates displayed angiogenic activity *in vitro *and *in vivo*, and synovia of arthritic paws appeared highly vascularised on histological examination, the functional capillary density in arthritic knee synovia was significantly decreased, whereas capillary diameter was increased. Of the 84 genes analysed, 41 displayed a differential expression in arthritic paws as compared to control paws. Most significant alterations were seen at the peak of clinical arthritis. Increased mRNA expression could be observed for VEGF receptors (*Flt-1, Flk-1, Nrp-1, Nrp-2*), as well as for midkine, hepatocyte growth factor, insulin-like growth factor-1 and angiopoietin-1. Signalling through NRP-1 accounted in part for the chemotactic activity for endothelial cells observed in arthritic paw homogenates. Importantly, therapeutic administration of anti-NRP1^B ^antibody significantly reduced disease severity and progression in CIA mice.

**Conclusions:**

Our findings confirm that the arthritic synovium in murine CIA is a site of active angiogenesis, but an altered balance in the expression of angiogenic factors seems to favour the formation of non-functional and dilated capillaries. Furthermore, our results validate NRP-1 as a key player in the pathogenesis of CIA, and support the VEGF/VEGF receptor pathway as a potential therapeutic target in RA.

## Introduction

Rheumatoid arthritis (RA) is an autoimmune disease that causes chronic inflammation of synovial joints, eventually resulting in the destruction of cartilage and bone [1]. During RA, the synovial tissue becomes infiltrated by inflammatory cells and increases greatly in mass due to the tumour-like proliferation of activated synoviocytes. In response to the growing metabolic demand caused by synoviocyte proliferation, blood vessels develop, which nourish and oxygenate this synovial pannus, promoting it to invade and degrade adjacent cartilage and bone. Neovessels exacerbate inflammation by further facilitating the ingress of inflammatory cells and mediators into the joint [2,3]. Targeting the synovial vasculature has therefore been proposed as a possible therapeutic strategy in RA, especially since the approval of angiogenesis inhibitors for certain cancers.

In RA, a luxuriant vasculature is an early feature of the arthritic synovium , and the number of synovial blood vessels correlates with hyperplasia, mononuclear cell infiltration and indices of joint tenderness [4]. The vascular turnover in the arthritic synovium is increased, and synovial endothelial cells express markers of proliferation [5]. Although the hyperplasic RA synovium is highly vascularised, paradoxically the tissue environment is chronically hypoxic [6]. Synovial fluids from RA joints have been shown to promote endothelial cell migration and proliferation, and to induce vessel formation in an angiogenesis assay [7,8], which reflects an active, pro-angiogenic phenotype of the arthritic synovium. Indeed, a number of angiogenic factors, expression of which is triggered by the hypoxic and inflammatory environment within the arthritic joint [9,10], are abundant in RA synovial tissue, including vascular endothelial growth factor (VEGF) [8,11], angiopoietins [10,12], hepatocyte growth factor (HGF) [13] and fibroblast growth factor (FGF)-2 [14]. Although new vessel formation is a highly coordinated process, VEGF is generally agreed to be a crucial regulator of angiogenesis in RA [3]. Increased amounts of VEGF can be detected in the synovial tissue and fluid as well as in the circulation of RA patients [9,11,15]. Serum levels of VEGF correlate with markers of inflammation, disease activity and radiographic progression [15,16]. During RA, VEGF seems to mediate its effects through its two tyrosine kinase receptors fms*-*like tyrosine kinase (FLT)-1 and kinase insert domain receptor (KDR; mouse homolog is fetal liver kinase (FLK)-1), and neuropilin (NRP)-1 [11,17].

However, although the importance of angiogenesis in arthritis progression is well recognized, there is little information about the function of the synovial vasculature as well as the molecular mechanisms implicated in arthritis-associated angiogenesis. Furthermore, the concomitant presence of hypoxia and angiogenesis is a conundrum. The model of collagen-induced arthritis (CIA) resembles many pathological features of RA, and although, it does not perfectly duplicate the human disease, it has helped to validate TNFα as a therapeutic target for RA [18]. In the mouse model of CIA, extensive synovial neovascularisation is a prominent histological feature of arthritic joints [19-21], and disease onset is associated with a reduction in synovial oxygen tension [19]. Synovial tissue isolated from arthritic paws of CIA mice induced a strong angiogenic response in a vascular window model, which was in part mediated through TIE-2 receptor signalling [20]. VEGF and its receptors, FLT-1 and FLK-1, are expressed within the inflamed synovial tissue of CIA joints, and local VEGF levels correlate with the degree of neovascularisation and disease severity [21]. Further, blocking VEGF function by administration of a neutralising VEGF antibody [22] or a soluble VEGF receptor (sFLT-1) [23] to mice with established CIA, suppressed disease progression, whereas exogenous VEGF exacerbated synovial inflammation and joint destruction [24].

Based on these observations, we utilised the mouse CIA model to investigate the functional capillary density in the inflamed synovium and to analyse genes that are involved in synovial angiogenesis during arthritis. Gene expression profiling of murine CIA has been conducted before [25,26]. However, these reports were based on a whole genome approach, and many angiogenesis-related genes, such as VEGF and its receptors, have not been assessed. Given that relatively little information is available on the kinetics of angiogenesis-relevant gene expression in CIA, the present study applied quantitative reverse transcription (RT)-PCR to investigate the expression of selected genes at onset, peak and declining phases of arthritis in DBA/1 mice. The hypoxic microenvironment in RA, coupled with the paradoxical feature of increased synovial vascularity, led us to speculate that the function of the synovial vasculature is disturbed and that abnormal angiogenic activity might contribute to the progression of CIA. We thus hypothesised that angiogenesis-related genes, including growth factors and receptors, would correlate with arthritis progression and angiogenic activity, particularly in the established phases of arthritis, allowing us to identify potential molecular targets for therapeutic intervention.

## Materials and methods

### Mice

Male DBA/1J (H-2^q^) and female C57BL/6 mice were purchased from Harlan Laboratories (Blackthorn, Bicester, UK) and maintained under standard conditions at the Biological Services Unit of the Kennedy Institute of Rheumatology, Imperial College London, UK. Studies were performed in accordance with the UK Animals (Scientific Procedures) Act 1986 regulations for the handling and use of laboratory animals, and followed an Ethics Committee and Home Office approved project licence (PPL No: 70/7335 Improving the therapy of rheumatoid arthritis). For intravital microscopy studies, male DBA/1J mice (10 weeks) were obtained from Taconic (Lille Skensved, Denmark) and kept under standard conditions at the animal care facility of the University of Rostock, Germany.

### Induction and evaluation of arthritis

Type II collagen (CII) was purified from bovine articular cartilage following the method described by Miller [27]. Prior to usage, CII was dissolved in 0.05M acetic acid. Complete Freund's adjuvant (CFA) was prepared by grinding 100 mg *Mycobacterium tuberculosis *(H37Ra; DIFCO, Becton Dickinson, Oxford, UK) and suspending it in 30 ml incomplete Freund's adjuvant (IFA, Becton Dickinson, Oxford, UK). For the induction of CIA, CII was emulsified in equal volumes of CFA to prepare a 1 mg/ml solution, and male DBA/1J mice aged 10-12 weeks were injected intradermally at the base of the tail with 100 μl. Mice were given a booster injection 21 days post-primary immunisation with 50 µg CII emulsified in IFA. A control group of mice received injections of CFA alone followed by IFA alone 21 days later. Following the boost, mice were examined daily for macroscopic signs and severity of arthritis. Arthritis severity was recorded for individual paws using the following scoring system: 0 = normal paw, 1 = mild oedema and/or erythema of the joint or individual digits; 2 = moderate oedema and erythema involving the entire paw, 3 = pronounced oedema and erythema accompanied with joint rigidity. An arthritis score was assigned to each mouse by summing up the individual scores, that is, a maximum score of 12. In conjunction with this, paw swelling was assessed by measuring the diameter of the hind paws using POCO 2T callipers (Krœplin Längenmesstechnik, Schlüchtern, Germany).

### Treatment of CIA with anti-NRP1^B ^antibody

Phage-derived monoclonal antibody against neuropilin-1 (anti-NRP1^B^), which recognises human and mouse NRP-1, was a kind gift from Genentech, Inc. (South San Francisco, CA, USA) [28]. To treat CIA, mice were injected intraperitoneally with 200 µg anti-NRP1^B ^antibody (n = 10) or PBS (n = 10) starting on day 1, following on day 4, and 7 of arthritis. In a pilot experiment, a group of mice received 200 µg human IgG1 isotype control antibody (Eureka, Emeryville, CA, USA). Arthritis scoring and paw swelling measurement were performed by an investigator blinded to treatment assignment.

### Immunohistological analysis

For immunohistological analysis, hind paws were removed post-mortem, cut once longitudinally and fixed overnight in 1% paraformaldehyde at 4°C. After decalcification in 0.3M EDTA for two weeks at room temperature, tissue was cryo-preserved, cut into sections of 6 µm thickness, and stained for CD31 according to standard procedures. Briefly, endogenous peroxidase activity was quenched by incubation in 0.3% H_2_O_2 _in methanol for 20 minutes. The specimens were blocked for 30 minutes in Tris buffered saline containing 10% normal rabbit serum (Dako UK Ltd, Ely, UK), and incubated overnight at 4°C with rat anti-mouse CD31 antibody (1/400; MEC13.3; Pharmingen, San Diego, CA, USA). The sections were subsequently incubated for 35 minutes at room temperature with biotinylated rabbit anti-rat IgG (1/400, Dako, Ely, UK), followed by 35 minutes at room temperature with avidin-biotin peroxidase complex and 5 minutes diaminobenzidine according to the manufacturer's instructions (Vectastain ABC kit and DAB, Vector Laboratories, Burlingame, CA, USA). Immunostained sections were counterstained with Harris haematoxylin. As control an isotope matched rat IgG2a antibody was used (R35-95, Pharmingen, San Diego, CA, USA), which showed no reactivity on tissue samples (data not shown). Histological scoring was performed blinded by one of the investigators.

### RNA extraction

At different time points during the course of CIA, mice were sacrificed, and paws were dissected and immediately flash frozen in liquid nitrogen. Paws were disrupted using a BioPulveriser® (BioSpec Products, Bartlesville, OK, US) and homogenised in RLT lysis buffer (Qiagen GmbH, Hilden, Germany) using a rotor-stator homogeniser (Ultra-Turrax T8; IKA Werke, Staufen, Germany). Total RNA was isolated from paw homogenates by phenol/chloroform extraction, followed by purification using the QIAamp® RNA Blood kit (Qiagen GmbH, Hilden, Germany) according to manufacturer's instructions. RNA quality was confirmed by spectrophotometry and gel electrophoresis. Samples were used for real-time RT-PCR or PCR-based array analysis as appropriate.

### Real-time RT-PCR

First strand cDNA was prepared using 500 ng of total RNA, random hexamer primers (Invitrogen, Renfrew, UK) and the Moloney murine leukaemia virus (MMLV) reverse transcriptase (Promega, Southampton, UK) according to the supplier's instructions. Real-time RT-PCR was carried out in a Rotorgene 6000 (Corbett Research, Sydney, Australia) using SYBR Green JumpStart Taq ReadyMix (SigmaAldrich, UK) and cDNA corresponding to 25 ng RNA. Cycling conditions were 2 minutes initial incubation at 50°C, followed by 5 minutes at 95°C and amplification for 40 cycles at 95°C for 15 seconds, 60°C for 30 seconds and 70°C for 40 seconds. The cycle threshold (Ct) value of each reaction was determined using Corbett's Rotor-Gene 6 software. To confirm primer specificity, amplicon melting curves were recorded after cycle 40 by heating from 60 to 95°C with a ramp speed of 0.5°C every second. Gene-specific mouse cDNA sequences were obtained from GenBank (NCBI), and primers were designed using the Primer3 design package hosted by the Whitehead Institute for Biomedical Research. Primers (Table 1) were designed to lie in different exons to prevent the amplification of genomic DNA, and optimised to work at an annealing temperature of 60°C. Primer amplification efficiencies were verified by real time RT-PCR on serial dilutions of cDNA according to Pfaffl [29]. The expression of target genes was expressed as mRNA level in fold change relative to the mRNA level in tissue of control mice after normalising to the *Arp *(acidic ribosomal phosphoprotein P0) gene using the comparative Ct-method (2^-ΔΔCT^).

**Table 1:** Primers applied in this study

Gene	Sense primer5'-3'	Antisense primer 5'-3'	Size (bp)	Reference
*Arp*	AATCTCCAGAGGCACCATTG	TTCAGCGTGTTCAGCAGTG	101	NM_007475
*Flt-1*	CGGCAGACCAATACAATCCT	CCGCTGCCTTATAGATGCTC	174	NM_010228
*Flk-1/Kdr*	CATGCACAGTCTACGCCAAC	CCTCCACGTGTCTCCATTCT	126	NM_010612
*Hgf*	GGCCATGGTGCTACACTCTT	CTTCTCCTTGGCCTTGAATG	132	NM_010427
*Igf-1*	TGGATGCTCTTCAGTTCGTG	GCAACACTCATCCACAATGC	113	NM_010512
*Il-1β*	CAGGCAGGCAGTATCACTCA	ATGAGTCACAGAGGATGGGC	140	NM_008361
*Il-6*	TACCACTCCCAACAGACCTGTC	CTGCAAGTGCATCATCGTTGTTC	140	NM_031168
*Mdk*	CCTGCAACTGGAAGAAGGAA	GAGGTGCAGGGCTTAGTCAC	166	NM_010784
*Nrp-1*	GGAGCTACTGGGCTGTGAAG	CCTCCTGTGAGCTGGAAGTC	134	NM_008737
*Plgf*	TGCTGGGAACAACTCAACAG	GGACACAGGACGGACTGAAT	146	NM_008827
*Tnf*	GACCCTCACACTCAGATCATC	CGCTGGCTCAGCCACTCCAGC	105	NM_013693
*Vegf120*	GGCTGCTGTAACGATGAAGC	GGCTTGTCACATTTTTCTGGC	184	NM_001025257
*Vegf164*	GGCTGCTGTAACGATGAAGC	GGCTCACAGTGATTTTCTGGC	184	NM_009505
*Vefg188*	GGCTGCTGTAACGATGAAGC	CTCACAGTGAACGCTCCAG	255	NM_001025250

Sequences represent primer pairs that were designed using Primer3 design package hosted by the Whitehead Institute for Biomedical Research [55]. References present Genbank accession numbers for each gene. All primer pairs were designed to span an intron to exclude potential genomic DNA contamination. (Arp*: *acidic ribosomal phosphoprotein P0; Flk-1: fetal liver kinase 1; Flt-1: fms-related tyrosine kinase 1; Hgf: hepatocyte growth factor; Igf-1: insulin-like growth factor; Il: interleukin; Mdk: midkine; Plgf: placental growth factor; Vegf: vascular endothelial growth factor)

### Mouse Angiogenesis RT^2^-Profiler™ PCR Array

The Mouse Angiogenesis RT^2^-Profiler™ PCR Array (APM-024; SuperArray Bioscience, Frederick, MD) profiles the expression of 84 genes involved in angiogenesis as well as five housekeeping genes (β-actin; glyceraldehyde 3-phosphate dehydrogenase, *Gapdh*; heat shock protein-1β, *Hsp-1*; hypoxanthine guanine phosphoribosyl transferase 1, *Hprt-1*; and β-glucuronidase, *Gusb*) by real-time PCR using the SYBR Green detection method. Therefore, 500 ng of total RNA was reverse transcribed using the ReactionReady™ First Strand cDNA Synthesis Kit (SABioscience, Frederick, MD, USA) following manufacturer's instructions. The generated cDNA was diluted with an appropriate volume of instrument-specific 2x SuperArray RT^2 ^Real-Time™ SYBR Green PCR Master Mix (PA-012) and ultra pure water, and 25 µl of this reaction mix was added to each well of the PCR array. The real-time PCR reaction was performed in an ABI 7700 thermocycler applying the following program: 2 minutes at 50°C, 10 minutes at 95°C and 40 cycles of 15 seconds at 95°C and 1 minute at 60°C. The ABI PRISM® 7700 Sequence Detection System was used to calculate the Ct value for each well. Data were normalised to four housekeeping genes and analysed by the comparative Ct-method (2^-ΔΔCT^).

### Preparation of paw homogenates

Mouse paws were skinned, snap frozen, pulverised, and homogenised in 1.5 ml ice-cold sterile PBS containing protease inhibitor cocktail (Roche Diagnostics, Burgess Hill, UK). Debris were removed by centrifugation at 4°C 13.000 rpm for 15 minutes and the supernatants were filtered using a 0.2 µm sterile syringe filter (Corning Incorporated, New York, USA). The protein content of the homogenates was determined using the Pierce BCA protein assay kit (Pierce, Rockford, IL, USA) following manufacturer's guidelines.

### Endothelial cell migration assay

Human microvascular endothelial cells (HMEC-1; gifted from Centers for Disease Control and Prevention, USA) were cultured in MCDB131 medium (Gibco, Invitrogen, Paisley, UK) containing 10% foetal calf serum (FCS; Biowest, Nuaillé, France), 100U/ml penicillin and 0.1 mg/ml streptomycin (PAA, Yeovil, UK) and 10 ng/ml human epidermal cell growth factor (PeproTech, London, UK) using gelatine-coated tissue culture flasks.

Migration of HMEC-1 in response to angiogenic stimuli was analysed using a modified Boyden chamber assay. Briefly, cell culture inserts (8.0 μm pore size; BD Biosciences, Oxford, UK) were placed into a 24-well plate and coated with gelatin. Recombinant human VEGF165 (20 ng/ml) or paw homogenates (100 ng/ml), prepared in MCDB131 medium containing 0.1% FCS were added to the lower wells. Prior to experiments, HMEC-1 were cultured overnight in 2% FCS MCDB131 medium. 100,000 cells in 200 μl of 0.1% FCS MCDB131 medium were added to the upper chambers and allowed to migrate for 5 hours at 37°C. After the migration period, cells were fixed with ice cold 70% ethanol for 10 minutes, treated with 0.1% TritonX-100 in PBS for 2 minutes and stained with haematoxylin for 10 minutes. Non-migrated cells were gently removed from the upper membrane surface using a cotton swab. Chemotaxis was quantified by counting nuclei in three random high power fields (HPF, 200×)/well. Each sample was assayed in triplicate.

### *In vivo *Matrigel plug assay

For this assay, 400 µl of ice-cold growth factor-reduced Matrigel (Becton-Dickinson, Bedford, MA, USA) were combined with 120 µg/ml paw homogenate, and injected subcutaneously into the back of female C57BL/6 mice (aged 7 weeks). Mice simultaneously received a plug containing either homogenate from a healthy or an arthritic paw. Seven days post-implantation, mice were sacrificed and plugs carefully dissected, photographed and weighted. To determine the angiogenic response, the haemoglobin content in the plugs was analysed using the tetramethylbenzidine method [30], and was normalised according to plug weight and expressed as ng/mg plug.

### Intravital microscopy (IVM)

Synovial capillaries in knee joints were monitored by IVM as previously described [31]. Studies were performed at the University of Rostock, Germany. CIA was induced as described above in male DBA/1J mice. A control group of mice received injections of CFA alone followed by IFA alone 21 days later. Mice were anesthetised with ketamine (90 mg/kg body weight) and xylacin (6 mg/kg), and kept on a heating pad (37°C). After excision of the skin and soft tissue surrounding the knee joint, the patella tendon was cut transversally and lifted to expose the Hoffa's fatty body. The tissue was superfused with 37°C saline and covered with a glass slide. Following a 10-minute stabilisation period, *in vivo *microscopy of the synovial tissue was performed. Mice received an intravenous injection of fluorescein isothiocyanate (FITC)-labelled dextran (15 mg/kg body weight; Sigma, Deisenhofen, Germany) for vascular contrast enhancement. *In vivo *microscopy was performed using a Zeiss microscope (Axiotech Vario 100HD; Carl Zeiss, Oberkochen, Germany) equipped with a 100W mercury lamp and filter sets for blue light epi-illumination (excitation/emission wavelength: 450 to 490 nm/> 520 nm). A 40-fold water immersion objective (total magnification: 630) was used to randomly select three to five non-overlapping regions of interest per tissue containing capillaries. Microscopic images were recorded for offline evaluation via a charge-coupled device video camera (FK 6990-IQ-S; Pieper, Schwerte, Germany). Quantitative offline analysis was performed by means of a computer-assisted image analysis system (CapImage v7.4; Dr. Zeintl Software, Heidelberg, Germany). Functional capillary density, defined as the total length of red blood cell-perfused capillaries per observed area, as well as capillary diameter were analysed.

### Statistical analysis

Data were analysed using Graph-Pad Prism 5.01 (GraphPad Software, San Diego, CA). Gene expression data were analysed applying the Students *t*-test, Mann-Whitney or one-way analysis of variance (ANOVA) as appropriate. Clinical arthritis data were analysed by two-way ANOVA. Histology data were analysed by the Mann-Whitney or chi-square test for trend as appropriate. *P*-values≤0.05 were considered significant.

## Results

### Synovial vascularisation in acute CIA

**Figure 1:**
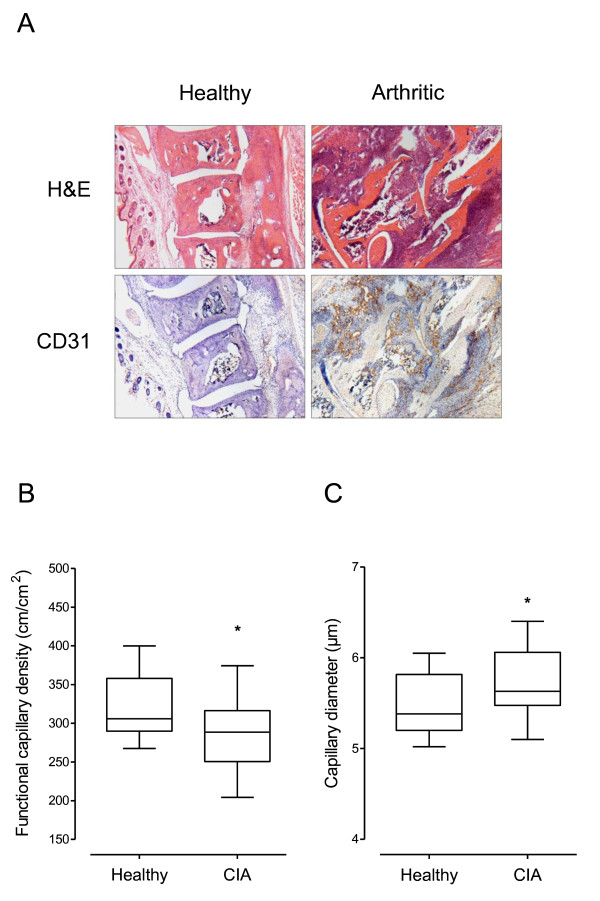
**Synovial neovascularisation in mice with acute collagen-induced arthritis (CIA)**. **A) **Histopathological features of haematoxylin and eosin (H&E) or anti-CD31 stained serial paw sections from healthy and arthritic mice at the peak of CIA. Images show the metatarsal joints (original magnification × 40). **B) **Functional capillary density and **C) **capillary diameter in knee joint synovial tissue of mice with established CIA (n = 29) in comparison to age-matched healthy (n = 17) mice as revealed by intravital microscopy (IVM). The arthritic group included mice analysed at different time points throughout the course of CIA. Data are expressed as mean ± SEM (Mann Whitney test, *P < 0.05).

Immunohistochemistry for CD31 was performed to assess the presence of microvessels in arthritic paws at the peak of CIA (Figure 1A). Staining for CD31 revealed occasional blood vessels with distinct lumen in paws obtained from healthy mice (not immunised with CII), whereas arthritic paws displayed marked vascularity especially in areas of dense cellular infiltration and in regions of synovium invading the bone. A number of vessels presented with a distinct lumen while other vessels appeared compressed. Although arthritic paw synovia showed regions of high vascular density, non-vascularised regions could also be identified.

Histology can help to detect synovial vessels, but it does not provide information about their functionality. We therefore applied intravital microscopy (IVM) to compare the functional capillary density in knee joints of healthy (not immunised with CII, n = 17) and arthritic (n = 29) mice at different time points throughout the disease course. The knee joint is the only joint which can be assessed for IVM without inducing major trauma [31]. Only mice with clinically evident knee joint arthritis were included in our analysis. Diseased knee joints presented with severe swelling of the soft tissue around the patella tendon, indicating synovial inflammation and oedema. The functional capillary density (FCD) was significantly decreased in arthritic tissue (*P *= 0.027, Mann Whitney test), with an FCD of 320.30 ± 9.76 cm/cm^2 ^(mean ± standard error of the mean, SEM) in knee synovia of healthy mice compared to an FCD of 286.70 ± 8.23 cm/cm^2 ^in arthritic mice (Figure 1B). The functional capillary density correlated inversely with the clinical score of analysed mice (Pearson correlation; *P *= 0.033, data not shown). The diameter of synovial capillaries was significantly (*P *= 0.030, Mann Whitney test) enlarged in arthritic joints compared to healthy control joints (Figure 1C; healthy 5.50 ± 0.08 µm *versus *arthritic 5.77 ± 0.07 µm; mean ± SEM). These data indicate that the vascular turnover is increased, but a fraction of capillaries are functionally abnormal in the arthritic synovial tissue during CIA.

### Arthritic paw homogenates induce endothelial cell migration *in vitro*

**Figure 2:**
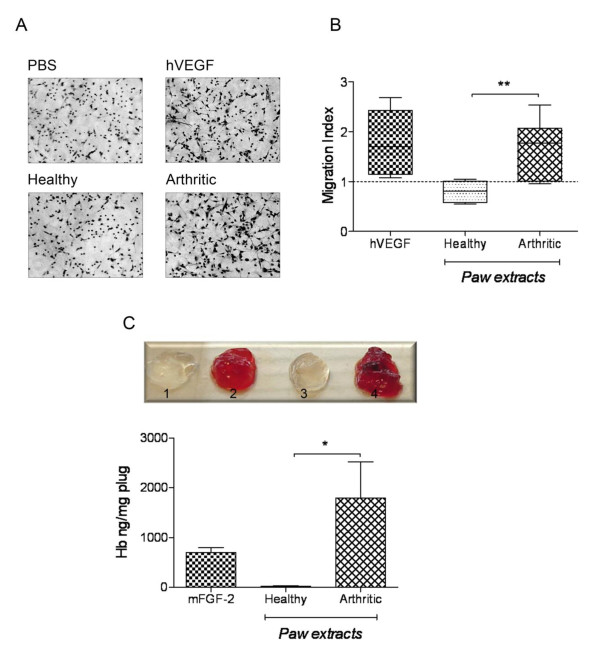
**Induction of angiogenic responses by joint homogenates from mice with collagen-induced arthritis (CIA)**. **A**) Arthritic joint homogenates significantly induced the migration of endothelial cells (HMEC-1) *in vitro*. Representative photographs of migrated HMEC-1 in response to different stimuli (original magnification × 200). **B**) Graph illustrates HMEC-1 migration in response to paw homogenates (100 µg protein/ml) of healthy (n = 5) or arthritic (n = 15) mice in comparison to 20 ng/ml recombinant human VEGF (hVEGF) (n = 6). The migration index was calculated by dividing the number of migrated cells in the presence of stimuli by the number of migrated cells in the presence of PBS (set as 1, dashed line). Results were expressed as mean ± SEM and analysed by unpaired *t*-test (**P < 0.01). **C**) Homogenates of arthritic paws induce angiogenesis *in vivo*. Representative images of Matrigel plugs comparing healthy (1, 3) with arthritic (2, 4) paw homogenate-containing plugs. Graph demonstrates the haemoglobin content of isolated Matrigel plugs. Recombinant murine fibroblast growth factor (FGF)-2 (mFGF-2, 30 ng/ml) was used as a positive control. Data expressed as mean ± SEM (healthy, n = 6; arthritic,n = 6; FGF-2, n = 3) and analysed by unpaired *t*-test: *P < 0.05.

Since synovial tissue of arthritic paws presented with increased vascularisation, as evident by abundant CD31 expression (Figure 1A), we tested whether angiogenic activity could be detected in paw homogenates of arthritic mice at the peak (day 8 following disease onset) of CIA. We therefore investigated the migratory response of HMEC-1 endothelial cells towards paw homogenates using a chemotaxis assay. Figure 2 (A, B) illustrates that homogenates of arthritic paws (n = 15) significantly induced the migration of HMEC-1 when compared to homogenates of healthy paws (n = 5) obtained from mice immunised with CFA/IFA without CII (*P *= 0.003). Arthritic paw homogenates elicited a similar migratory response to that induced by hVEGF (migratory index 1.66 ± 0.14 and 1.77 ± 0.26, respectively), whereas healthy paw homogenates displayed a slightly lower migratory activity than PBS.

### Arthritic paw homogenates induce angiogenesis *in vivo*

To investigate whether paw homogenates were able to elicit an angiogenic response *in vivo*, Matrigel plug assays were performed. For this purpose, Matrigel containing paw homogenates at a concentration of 120 µg protein/ml Matrigel was injected subcutaneously into the backs of C57BL/6 mice. Each animal simultaneously received one plug containing homogenate from a healthy paw and one plug containing homogenate from an arthritic paw (or murine FGF-2 as a positive control). Plugs were removed 7 days later. The degree of plug vascularisation was analysed by determining the haemoglobin content of the implants. Plugs with arthritic tissue homogenates (n = 6) (peak of CIA, day 8) contained significantly (*P *= 0.034) more haemoglobin than plugs with healthy (n = 6) tissue extracts (Figure 2C; healthy 20.98 ± 13.27 *versus *arthritic 1793 ± 723.6 ng haemoglobin/mg plug; mean ± SEM).

### Temporal expression profile of angiogenesis-relevant genes during CIA

In order to determine if the expression of genes regulating angiogenesis was altered during the course of CIA, age-matched, male DBA/1 mice were immunised with CII. Arthritic paws were collected on day 1 (onset), day 4, day 8 (peak) and day 12 (declining phase) of CIA. To account for possible adjuvant-induced expression changes, one group of mice was immunised with CFA/IFA alone without CII, while a further group of mice was left untreated (naïve). For the Angiogenesis RT^2^-Profiler® Array, a pool of RNA was prepared for each control and time point, containing the same amount of RNA from whole paws of different animals (n = 4). Expression levels were analysed from six different groups: naïve mice, mice immunised with CFA/IFA without CII (healthy), arthritic animals on day 1, 4, 8 and 12 of CIA. The mRNA level of target genes was normalised to the mean mRNA level of four housekeeping genes (β-actin, *Gapdh, Hsp-1, Hprt-1*), since their expression was stable between sample groups. The expression of β-glucuronidase (*Gusb*) was increased in arthritic samples and therefore excluded from the analyses. Indeed, it has been reported that the level of GUSB is elevated in synovial fluids of RA patients [32]. The immunisation with CFA/IFA alone without CII resulted in slight alterations in the expression of genes for pro-inflammatory cytokines (*Il-6, Tnfα*), chemokines (*Cxcl5, Cxcl1*) and leptin (data not shown) when compared to naïve animals. Therefore, all subsequent gene expression in arthritic paws was compared using the gene expression in paws of mice immunised with CFA/IFA without collagen (healthy) as baseline.

The RT^2^-Profiler® Array profiles 84 angiogenesis-relevant genes, of which 41 were identified as altered (fold change ≤ -2 or ≥ 2) at least at one time point of CIA (Table 2). Among these genes, 32 were up-regulated and 9 were down-regulated in arthritic paws compared to control paws. The majority of up-regulated genes reached their maximum expression on day 8 of arthritis, at which time symptoms of arthritis peaked, with gene expression levels gradually decreasing again on day 12, when the disease started to resolve, indicating a role for those genes in disease progression.

**Table 2:** Angiogenesis-related genes altered during the course of acute CIA

Gene	Gene bank	Gene name	Onset		Day 4		Day 8		Day 12	
			**Fold change**	***P-value***	**Fold change**	***P-value***	**Fold change**	***P-value***	**Fold change**	***P-value***

***Growth factors and receptors***										
*Ang-1*	NM_009640	Angiopoietin 1	1.3		1.8		2.7	*0,004*	1.5	
*Edg1*	NM_007901	Endothelial differentiation sphingolipid G-protein-coupled receptor 1	1.8		2.4	*0,001*	3.3	*0,002*	2.4	*0,005*
*Egf*	NM_010113	Epidermal growth factor	-1.4		-2.9	*0,013*	-3.7	*0,005*	-2.9	*0,005*
*Ereg*	NM_007950	Epiregulin	-1.4		-2.1	*0,001*	-2.3	*0,002*	-1.8	
*Fgf-1*	NM_010197	Fibroblast growth factor 1	-1.3		-2.3	*0,004*	-1.7		-1.1	
*Flk-1*	NM_010612	Fetal liver kinase	1.3		2.2	*0,011*	3.0	*0,006*	1.9	
*Hgf*	NM_010427	Hepatocyte growth factor	2.2	*0,006*	4.6	*0,001*	6.0	*0,001*	3.9	*0,002*
*Igf-1*	NM_010512	Insulin-like growth factor 1	1.4		2.4	*0,000*	4.1	*0,001*	2.4	*0,000*
*Lep*	NM_008493	Leptin	-2.5	*0,007*	-5.8	*0,002*	-6.8	*0,002*	-3.9	*0,015*
*Mdk*	NM_010784	Midkine	1.5		2.4	*0,008*	7.5	*0,002*	5.3	*0,003*
*Nrp-2*	NM_010939	Neuropilin 2	1.4		2.0	*0,009*	2.4	*0,015*	2.2	*0,011*
*Tgfβ1*	NM_011577	Transforming growth factor, beta 1	1.6		2.1	*0,009*	2.3	*0,005*	1.8	
*Tgfβ2*	NM_009367	Transforming growth factor, beta 2	-1.4		-2.0	*0,021*	-1.3		1.1	
*Tgfβr1*	NM_009370	Transforming growth factor, beta receptor I	1.5		1.6		2.0	*0,003*	1.5	
***Proteases and inhibitors***										
*Mmp-2*	NM_008610	Matrix metallopeptidase 2	1.7		2.6	*0,001*	4.1	*0,000*	3.0	*0,001*
*Mmp-9*	NM_013599	Matrix metallopeptidase 9	3.4	*0,002*	16.2	*0,001*	20.2	*0,001*	12.7	*0,001*
*Mmp-19*	NM_021412	Matrix metallopeptidase 19	3.6	*0,001*	4.6	*0,002*	5.2	*0,000*	3.8	*0,003*
*Timp-1*	NM_011593	Tissue inhibitor of metalloproteinase 1	17.1	*0,009*	34.0	*0,001*	24.4	*0,001*	11.6	*0,001*
*Timp-2*	NM_011594	Tissue inhibitor of metalloproteinase 2	1.2		1.4		2.4	*0,001*	2.1	*0,001*
***Adhesion molecules***										
*Cdh5*	NM_009868	Cadherin 5	1.6		3.0	*0,003*	3.5	*0,002*	2.3	*0,017*
*Eng*	NM_007932	Endoglin	1.6		1.9		2.6	*0,007*	1.9	
*Itgβ3*	NM_016780	Integrin beta 3	1.9		5.9	*0,001*	6.1	*0,001*	3.4	*0,001*
*Pecam1*	NM_008816	Platelet/endothelial cell adhesion molecule 1	1.7		1.7		2.1	*0,012*	1.8	
*Stab1*	NM_138672	Stabilin 1	2.6	*0,000*	4.0	*0,001*	3.3	*0,001*	2.0	*0,002*
***Transcription factors***										
*Efnβ2*	NM_010111	Ephrin B2	-2.0		-2.5	*0,001*	-1.7		-1.1	
*Hif-1α*	NM_010431	Hypoxia inducible factor 1, alpha subunit	1.5		2.6	*0,001*	3.8	*0,000*	1.6	
*Tbx1*	NM_011532	T-box 1	-1.7		-3.3	*0,003*	-3.3	*0,011*	-2.0	*0,013*
*Tnfαip2*	NM_009396	Tumor necrosis factor, alpha-induced protein 2	2.0	*0,002*	2.6	*0,004*	2.6	*0,001*	2.0	*0,001*
***Anti-angiogenic factors***										
*Tsp-1*	NM_011580	Thrombospondin 1	1.8		1.9		2.8	*0,000*	2.0	*0,006*
*Tsp-2*	NM_011581	Thrombospondin 2	1.3		2.2	*0,003*	2.9	*0,001*	2.0	*0,002*
*Col4α3*	NM_009929	Procollagen, type IV, alpha 3/tumstatin	1		-2.0	*0,015*	-2.0	*0,011*	-1.3	
*Col18α1*	NM_007734	Procollagen, type XVIII, alpha 1/endostatin	1.1		1.7		2.0	*0,012*	2.0	*0,015*
***Cytokinesand chemokines***										
*Il-1β*	NM_008361	Interleukin 1 beta	34.1	*0,000*	48.2	*0,000*	60.0	*0,000*	16.2	*0,000*
*Il-6*	NM_031168	Interleukin 6	183.7	*0,000*	190.4	*0,000*	164.7	*0,000*	30.3	*0,001*
*Tnfα*	NM_013693	Tumor necrosis factor	2.2	*0,014*	2.2	*0,009*	3.7	*0,010*	2.2	*0,018*
*Ccl2*	NM_011333	Chemokine (C-C motif) ligand 2/MCP1/HC11	15.0	*0,000*	11.0	*0,001*	10.0	*0,000*	3.0	*0,001*
*Cxcl1*	NM_008176	Chemokine (C-X-C motif) ligand 1/Gro1	19.2	*0,003*	35.7	*0,001*	21.0	*0,001*	7.0	*0,019*
*Cxcl2*	NM_009140	Chemokine (C-X-C motif) ligand 2/Grob	8.7	*0,001*	13.4	*0,001*	15.2	*0,001*	6.1	*0,001*
*Cxcl5*	NM_009141	Chemokine (C-X-C motif) ligand 5/ENA-78	126.3	*0,002*	378.4	*0,001*	345.8	*0,001*	70.3	*0,003*
*Ccl11*	NM_011330	Small chemokine (C-C motif) ligand 11/eotaxin	1.1		-2.0	*0,003*	-2.0	*0,019*	1.2	
*Csf3*	NM_009971	Colony stimulating factor 3 (granulocyte)/G-CSF	86.9	*0,016*	69.7	*0,017*	48.9	*0,020*	20.3	*0,034*

Angiogenesis RT^2^-Profiler PCR arrays (APM-024; SuperArray Bioscience, Frederick, MD) were performed using cDNA from paws of CIA mice at different time points post-arthritis onset. For each time point, RNA from four animals was pooled and analysed in duplicate. Data are expressed as fold change relative to the expression in paws from healthy mice after normalisation to four housekeeping genes. The table lists genes that exhibit at least a two-fold difference in expression at one or more time-points in arthritic tissue when compared to control tissue. Data were analysed by paired *t*-test.

As might be predicted, a substantial fold increase could be observed for genes related to inflammation (for example, *Cxcl5, Cxcl1, Il-6, Il-1*), cell adhesion (for example, integrin β3, cadherin 5) and proteolysis (for example, *Mmp-9, Mmp-19*). Up-regulated angiogenic growth factors included *Hgf*, insulin-like growth factor (*Igf*)-1, midkine *(Mdk) *and angiopoietin (*Ang*)-1, whereas leptin and epidermal growth factor (*Egf*) were decreased in arthritic paws. No difference for total *Vegf/a *transcript levels could be detected between arthritic and control tissue (not shown), whereas its receptors *Flk-1 *and neuropilin (*Nrp*)-2 were increased in arthritic mouse paws. Interestingly, the expression levels of genes that play a role in inhibiting angiogenesis were also increased during CIA (for example, thrombospondins, endostatin).

**Figure 3:**
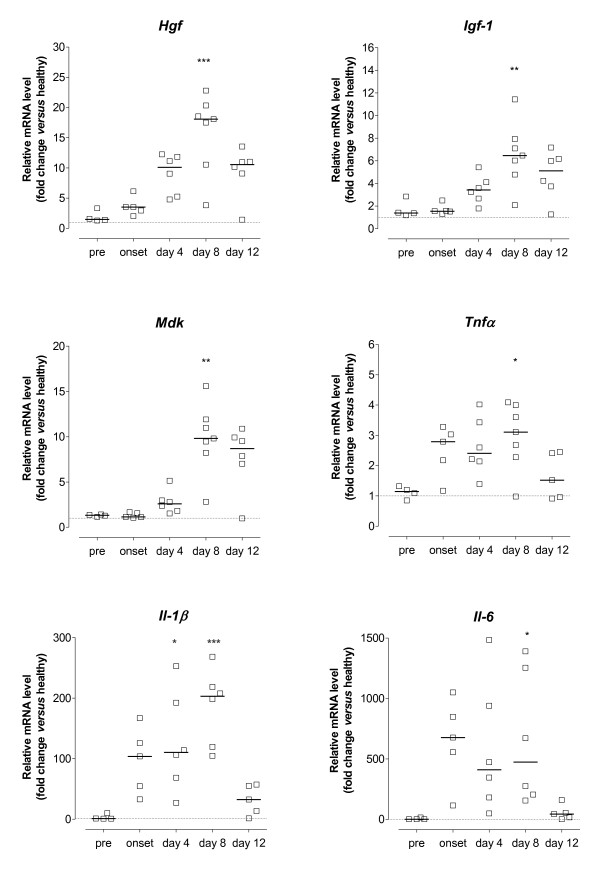
**mRNA expression of angiogenesis-modulating factors during the course of collagen-induced arthritis (CIA)**. Mice at different stages of disease were sacrificed and whole paws processed for mRNA quantification by real-time RT-PCR. Data are presented as fold change in mRNA level relative to the mean expression in six age-matched healthy controls (dotted line) after normalisation to the acidic ribosomal phosphoprotein P0 (*Arp*) gene. Data are from individual mice (n = 5 to 7), bars indicate the median mRNA level of the different groups. Statistical analysis of data was performed using one-way ANOVA with Dunn's post hoc test for multiple comparisons, compared against pre-arthritic mice (*P < 0.05, **P < 0.01, ***P < 0.001).

Gene-specific real-time RT-PCR for selected genes using samples from individual animals was performed to validate the observed trends in the array. For this purpose, samples were obtained from arthritic animals on day 1 (onset, n = 5), day 4 (n = 6), day 8 (n = 7) and day 12 (n = 6) of CIA, as well as from mice immunised with CFA/IFA without CII (healthy, n = 6). To investigate possible alterations before the onset of clinical arthritis, we included a group of mice that were sacrificed 14 days after immunisation with collagen but without macroscopic signs of CIA (pre-arthritic, n = 4). The expression of target genes was normalised to *Arp *expression, which displayed expression stability among the different conditions (healthy, pre-arthritic, arthritic). Figure 3 illustrates the kinetics of mRNA expression for *Hgf, Igf-1*, *Mdk*, *Tnfα*, *Il-1β *and *Il-6 *in arthritic tissue. Their temporal expression pattern reflected disease activity, with most prominent changes occurring at the peak phases of CIA, with mean clinical scores ± SEM for the analysed paw of 1 ± 0.00 on day 1, 1.75 ± 0.25 on day 4, and 2.14 ± 0.37 on day 8. A significant increase in transcript levels could be observed on day 8 for all genes, with mRNA levels gradually decreasing on day 12 (mean clinical score ± SEM of the analysed paws 1.67 ± 0.38), which is consistent with the array results. We have previously reported that expression of *Ang-1*, *Ang-2*, tyrosine kinase with immunoglobulin-like and EGF-like domains (*Tie*)-1 and *Tie-2 *increases as arthritis progressed, peaking on day 8 of disease [33]. Of these, *Ang-2 *and *Tie-2 *showed modest changes (below 2-fold) on the array, whereas *Ang-1 *increased 2.7-fold on day 8 (Table 2).

**Figure 4:**
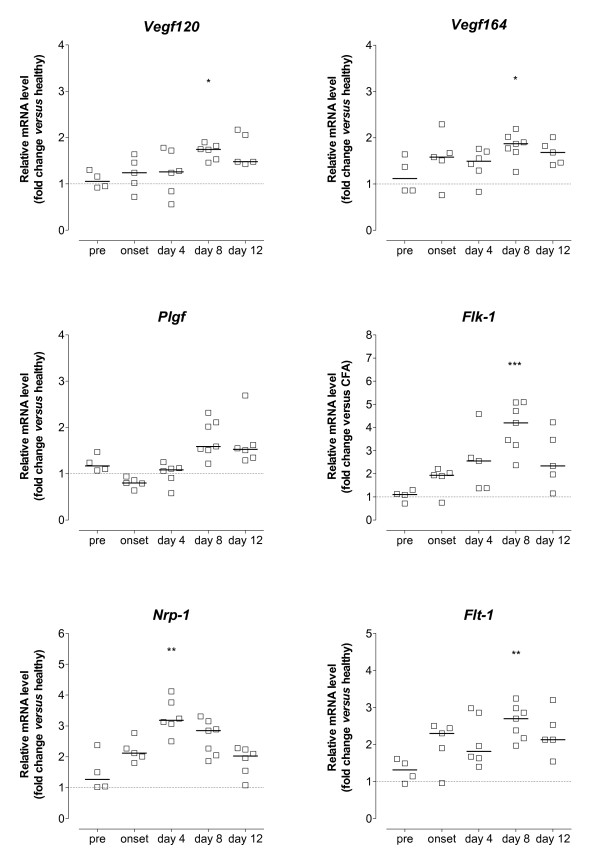
**mRNA expression of the VEGF/VEGF receptor family during the course of CIA**. Mice at different stages of disease were sacrificed and whole paws processed for mRNA quantification by real-time RT-PCR. Data are presented as fold change in mRNA level relative to the mean expression in six age-matched healthy controls (dotted line) after normalisation to the acidic ribosomal phosphoprotein P0 (*Arp*) gene. Data are from individual mice (n =5 to 7), bars indicate the median mRNA level of the different groups. Statistical analysis of data was performed using one-way ANOVA with Dunn's post hoc test for multiple comparisons, compared against pre-arthritic mice (*P < 0.05, **P < 0.01, ***P < 0.001).

Unexpectedly, the array identified the expression of total *Vegf/a *mRNA as unchanged in arthritic tissue, despite strong evidence supporting a role for VEGF in both animal models of disease [21-23] and human RA [9,11,15]. Therefore, we examined the expression of different VEGF isoforms using samples from individual animals by gene-specific RT-PCR (Figure 4). Transcript levels for *Vegf120 *and *Vegf164 *were significantly elevated on day 8 of arthritis compared to pre-arthritic animals. However, the changes were only minor (below 2-fold). The expression of *Vegf188 *did not change during the course of CIA (data not shown). No significant change in the mRNA level for placental growth factor (*Plgf*) could be observed at the analysed time points. For the regulation of angiogenesis, not only the expression of growth factors is important, but also the expression of the respective receptors. Maximal induction of *Flt-1 *and *Flk-1 *transcript levels was observed on day 8 (2.7- and 4.2-fold respectively), whereas the level for *Nrp-1*, which can act as an enhancer for VEGF receptor signalling, had its peak expression already on day 4 of CIA. This confirms array results, although changes identified by the array for *Flt-1 *and *Nrp-1 *mRNA were below 2-fold.

### Anti-NRP1^B ^inhibits HMEC-1 migration induced by arthritic paw homogenates

**Figure 5:**
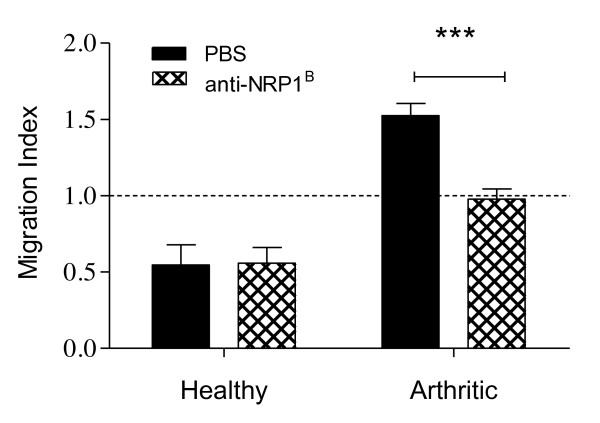
**Blocking neutropilin (NRP)-1 inhibits human microvascular endothelial cells (HMEC)-1 migration induced by arthritic paw homogenates**. The ability of anti-NRP1^B ^antibody to inhibit the migration of HMEC-1 in response to paw homogenates was evaluated using a chemotaxis assay. Cells were seeded in presence of anti-NRP1^B ^(10 µg/ml) or PBS into the upper chamber and allowed to migrate towards paw homogenates (100 µg protein/ml) in the lower chamber. PBS as stimulant was used as a negative control (set as 1, dashed line). Data expressed as mean ± SEM (healthy, n = 3; arthritic, n = 6). Data were analysed using two-way ANOVA: ***P < 0.001.

Our gene expression analysis revealed that the mRNA level for *Nrp-1 *is elevated in arthritic tissue of mice with CIA. As opposed to the other VEGF receptors, it reached its maximum expression already on day 4 of arthritis. To further analyse its role in arthritis-induced angiogenesis, we investigated whether NRP-1 function is required for the induction of endothelial cell migration by arthritic paw homogenates applying a neutralising antibody that specifically blocks the binding of VEGF121 and VEGF164 to NRP-1 [28,34]. Therefore, HMEC-1 were added together with either anti-NRP1^B ^or PBS into the upper chamber of the cell culture inserts, while paw homogenates (100 µg protein/ml) were added into the bottom cell culture well to promote endothelial cell migration. Blocking the VEGF-binding domain of the NRP-1 receptor on endothelial cells significantly reduced endothelial cell migration towards arthritic paw homogenates (Figure 5). However, the migratory response was still greater than induced by healthy paw homogenates, which seem to inhibit endothelial migration, as the number of migrated cells was lower than that induced by PBS alone, which is consistent with data presented in Figure 4B.

### Treatment with anti-NRP1^B ^significantly reduces disease severity and joint destruction in collagen-induced arthritis

**Figure 6:**
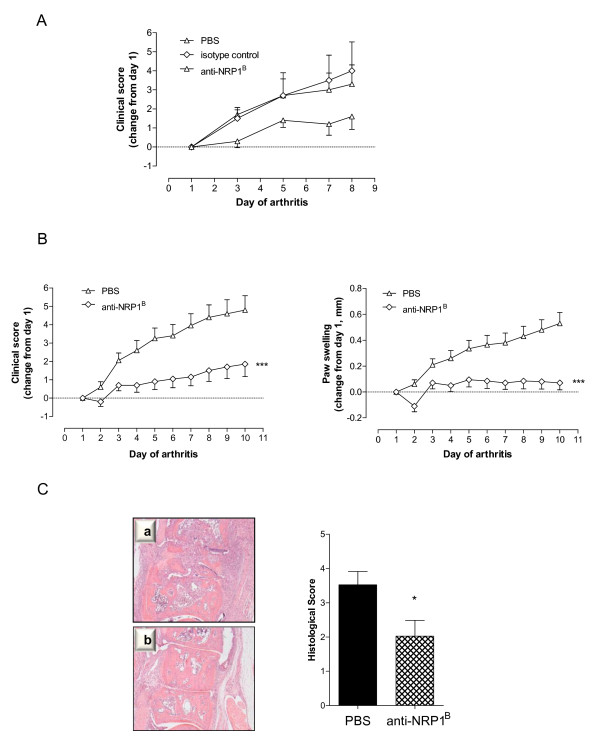
**Treatment with phage-derived monoclonal antibody against neuropilin-1 (anti-NRP1^B^) reduces disease severity in collagen-induced arthritis (CIA)**. **A) **Isotype-matched control antibody has no effect on disease progression in CIA mice. Starting at the onset of clinically evident arthritis, mice were randomised into three groups (n = 5/group) and treated intraperitoneally with 200 µg anti-NRP1^B ^antibody, 200 µg isotype-matched control antibody or PBS on day 1, day 4 and day 7 of CIA. The clinical score was recorded every other day and presented as change from day 1. **B) **Effect of anti-NRP1^B ^on the clinical progression of arthritis. Starting on the first day of clinically evident arthritis, mice received 200 µg anti-NRP1^B ^antibody or PBS intraperitoneally on day 1, day 4 and day 7 of CIA. The clinical score and paw swelling were recorded daily and presented as change from day 1. Data are expressed as mean ± SEM (n = 10/group) and analysed by two-way ANOVA (PBS *versus *anti-NRP1^B^; ***P < 0.001). Results are representative of three independent experiments. **C) **Paws from anti-NRP1^B^-treated mice (b) displayed significantly reduced articular inflammation and destruction compared to those from PBS-treated mice (a). After treatment, hind paws were collected, fixed, decalcified, and haematoxylin/eosin sections prepared. Histological appearance was scored for the degree of bone erosion, synovial hyperplasia, and cellular infiltration. Shown are representative metatarsal joint sections from PBS-treated and anti-NRP1^B^-treated mice (original magnification × 40). The histological changes differed significantly between the two treatment groups (Mann Whitney test, *P < 0.05).

Since anti-NRP1^B ^reduced the migratory response of endothelial cells induced by arthritic paw homogenates, we aimed to further elucidate the role for NRP-1 in disease progression in arthritic animals. In a pilot study, a small cohort of mice (n = 5 per group) received intraperitoneal injections of 200 µg anti-NRP1^B^, 200 µg isotype matched control antibody or an equivalent volume of PBS on day 1 (onset - when first clinical signs of CIA were evident), and additionally on day 4 and day 7 of arthritis. Mice were scored every other day until day 8 of arthritis. Treatment with anti-NRP1^B ^resulted in a marked attenuation of CIA, with the clinical score significantly lower when compared to both untreated mice (*P *< 0.01, two-way ANOVA) and isotype control antibody-treated mice (*P *< 0.01), thus ruling out unspecific effects (Figure 6A).

Subsequently, a larger cohort of mice (n = 10 per group) received intraperitoneal injections of 200 µg anti-NRP1^B ^or an equivalent volume of PBS on day 1 (onset - when first clinical signs of CIA were evident), and additionally on day 4 and day 7 of arthritis. Over the course of ten days, mice were macroscopically monitored and assigned a clinical score. Additionally, paw swelling of hind paws was measured. In order to normalise the results, data were calculated as change relative to day 1. Treatment with anti-NRP1^B ^resulted in a marked attenuation of CIA. As Figure 6B demonstrates, the administration of the anti-NRP1^B ^antibody significantly reduced the severity of paw inflammation indicated by the change in clinical score compared to PBS administration (*P *< 0.001). For instance, the change in clinical score on day 10 of arthritis after anti-NRP1^B ^treatment was 1.85 ± 0.67 (mean ± SEM) compared to 4.8 ± 0.79 for PBS-treated mice. The increase in hind paw swelling following anti-NRP1^B ^treatment was also significantly lower when compared to mice receiving only PBS treatment (*P *< 0.001). To illustrate, on day 10 the change in paw swelling in anti-NRP1^B^-treated mice was 0.07 ± 0.05 mm (mean ± SEM) and 0.53 ± 0.08 mm in PBS-treated mice.

To determine whether anti-NRP1^B ^antibody ameliorated joint destruction, hind paws were harvested on day 10 and histologically examined. Joints of mice treated with anti-NRP1^B ^exhibited fewer infiltrating inflammatory cells in the synovium as well as reduced cartilage erosion and bone degradation when compared to those of mice treated with PBS alone (Figure 6C). The mean histological score was significantly lower than after PBS administration (*P *= 0.041). Overall, anti-NRP1^B^-treated mice exhibited a significant improvement in joint histology (Table 3). The percentages of mice exhibiting normal joint histology or only mild synovitis were 42.1% and 17.5%, respectively, compared to 20.7% and 5.2% in PBS-treated mice. Taken together, these results demonstrate that blocking VEGF binding to NRP-1 reduces clinical and histopathological severity of CIA.

**Table 3:** Effect of anti-NRP1^B ^treatment on joint inflammation and destruction in mice with collagen-induced arthritis

	Joint findings, %				
	
Treatment	Normal	Mild	Moderate	Severe	*P†*
PBS	20.7	5.2	32.7	41.4	
anti-NRP1^B^	42.1	17.5	22.9	17.5	0.0021

Values are the percentage of joints with normal appearance or exhibiting inflammation and cartilage/bone degradation graded as mild, moderate, or severe, as assessed by haematoxylin and eosin. The numbers of joints assessed were as follows: 57 in the phage-derived monoclonal antibody against neuropilin-1 (anti-NRP1^B^)-treated group and 58 in the PBS-treated group.

*† *The absolute numbers of mice exhibiting normal, mild, moderate, and severe arthritis changes were compared, and significance versus untreated mice was analysed by chi-square test for trend.

## Discussion

In RA and the relevant mouse CIA model, angiogenesis is considered to promote and exacerbate synovial inflammation and cartilage/bone erosion by facilitating the recruitment of inflammatory cells, and by providing oxygen and nutrients to the hyperplasic tissue [2,3]. Although the importance of angiogenesis in the pathology of arthritis is well recognised, there is little information about the function of the blood vasculature. In the present study, we demonstrated for the first time that despite an increased vascular turnover, the functional capillary density is decreased in synovial tissue of arthritic joints during CIA. This reduction will further exacerbate the insufficient supply of nutrients and oxygen to the synovium, which could explain the observed hypoxic environment found in arthritic joints of mice [19]. Additionally, we observed an active pro-angiogenic microenvironment in arthritic paws, which was also reflected in an increased number of CD31-positive vessels and endothelial cells. These findings suggest that an imbalance of molecular factors regulating vessel formation and maturation contributes to abnormal synovial neovessel function.

To further elucidate the molecular mechanisms underlying this dysregulated synovial angiogenesis and to identify potential targets for therapeutic intervention, we characterised the expression of angiogenesis-related genes during the progression of murine CIA applying quantitative real-time RT-PCR. Our results show that the expression of hypoxia inducible factor 1alpha (HIF-1α), a key mediator of hypoxic responses, is elevated during the course of CIA in arthritic paws compared to healthy paws. In RA, HIF-1α induces the transcription of matrix metalloproteinases (MMPs) and pro-angiogenic growth factors, including VEGF, thus activating the angiogenic cascade as well as matrix degradation [35]. Not surprisingly, the most notable alterations occurred in the expression of genes involved in inflammation (for example, pro-inflammatory cytokines *Il-1β*, *Il-6*, *Tnf*α) and tissue remodelling (for example, *Mmp-2*, *Mmp-9*, *Mmp-19*). TNFα and IL-1β, as well as playing roles in the inflammatory component of RA, modulate synovial angiogenesis by inducing the production of angiogenic mediators like VEGF, ANG-1, ANG-2 and TIE-2 by RA synoviocytes [9,10,20]. MMPs participate in angiogenesis by degrading and remodelling the extracellular matrix and basement membranes, allowing activated endothelial cells to proliferate and migrate, as well as releasing extracellular matrix-bound growth factors such as FGF-2, VEGF or IGF-1 [36].

Our results further indicate that a hypoxic and pro-inflammatory microenvironment induces the transcriptional activation of angiogenic growth factors in arthritic joints of CIA mice, including midkine and *Hgf*. Elevated levels of midkine have been detected in the serum and synovial fluid of RA patients [37,38], and HGF levels within the arthritic joint correlate with disease activity [39] and synovial microvessel density [13]. On the other hand, EGF, which has been reported as elevated in RA synovial fluids [40], was down-regulated during CIA. Further, we detected a significant decrease of leptin mRNA levels in arthritic paws, which is consistent with published gene expression data in murine CIA [41]. Leptin, apart from the regulation of food intake, has been implicated in the regulation of immune responses and angiogenesis [42].

Although the mRNA levels of VEGF isoforms and *Plgf *were only modestly (below 2-fold) altered during CIA, the VEGF signalling pathway was clearly affected, since the expression of its tyrosine kinase receptors *Flt-1 *and *Flk-1 *as well as its co-receptors *Nrp-1 *and *Nrp-2 *was significantly increased during CIA. This relatively small increase in *Vegf *expression was unexpected, especially in view of data showing high VEGF secretion by mouse CIA synovial membrane cells [23] or the effectiveness of anti-VEGF treatment in experimental arthritis [21-23]. However, the difference in VEGF mRNA levels [43] or protein [44] levels between non-arthritic and arthritic tissue was also reported to be not more than 1.5- to 1.6-fold. Taken together, these findings indicate that in the CIA model, VEGF expression does not change markedly, but seems nonetheless sufficient to induce pathological angiogenesis, and suggest that VEGF-mediated angiogenesis is largely modulated through an increase in surface expression of VEGF receptors on target cells, thereby increasing their responsiveness towards the angiogenic stimuli.

Another pathway involved in vessel formation and maturation is the ANG/TIE system. In a previous study we have shown that the expression of *Ang-1*, *Ang-2*, *Tie-1 *and *Tie-2 *mRNA is significantly higher in arthritic tissue of CIA mice, and that the application of a splice variant of TIE-1 ameliorates disease severity, reduces synovial vascularisation and bone destruction [33]. Our angiogenesis array identified *Ang-1 *as up-regulated by more than 2-fold, whereas *Ang-2 *was increased by only 1.6-fold on day 8 of CIA. Co-expression of angiopoietins and their receptors is observed in rheumatoid synovial tissue [10,12]. ANG-1 signalling through TIE-2 maintains vessel integrity through the recruitment of pericytes, while ANG-2 blocks ANG-1/TIE-2 signalling, loosening the vascular structure and exposing the endothelium to inducers of angiogenesis such as VEGF [45]. In addition to increased transcription of angiogenic factors, the mRNA levels of some anti-angiogenic molecules (thrombospondins, endostatin) were also elevated, although their expression might not be sufficient enough to counteract the angiogenic response, since arthritic paw homogenates were able to induce endothelial cell chemotaxis *in vitro *as well as angiogenesis *in vivo*.

Our results reveal the existence of angiogenesis-related gene expression signatures in arthritic tissue and clearly indicate an active pro-angiogenic microenvironment in arthritic paws, which was also reflected in the increased number of CD31 positive vessels and endothelial cells. However, although the vascular turnover was increased within the inflamed synovium during CIA, the functional capillary density in arthritic synovial tissue was decreased. A previous IVM study revealed a trend towards a reduced functional capillary density in the synovium of pre-arthritic knee joints already before the onset of CIA [26]. Further, the diameter of the synovial capillaries in pre-arthritic mice was enlarged. These findings are in agreement with our observations in arthritic mice. It may seem counterintuitive that the arthritic synovium exhibits a lower functional microvessel density than healthy synovium, despite exhibiting a pro-angiogenic microenvironment and histologically an increased vascularisation. The human RA synovium contains regions of ongoing angiogenesis, indicated by endothelial cell proliferation, and regions of vascular regression, evident by markers of DNA damage and endothelial cell death [5]. However, angiogenesis not only depends on endothelial cell invasion and proliferation, but also requires coverage of vascular sprouts by peri-endothelial cells for vessel stabilization. Similar to tumour vasculature, a significant fraction of the synovial vasculature in arthritic joints of RA patients has not yet recruited peri-endothelial cells, which are essential for functional vascular patterning, diameter regulation, and vessel stabilisation [46,47].The vascular network is thus dysfunctional in RA, and the presence of unstable vessels is associated with hypoxia, incomplete endothelial cell-pericyte interactions, increased DNA damage, disease progression and increased lymphocyte infiltration [46,47]. Over-expression of angiogenic factors and receptors, as demonstrated in our present study, is likely to drive and maintain angiogenesis, as evidenced by the pro-angiogenic activities of arthritic paw extracts, but if uncontrolled, might actually promote further hypoxia and hence angiogenesis, while not allowing vessel maturation. For example, VEGF-mediated vessel formation has been associated with an imbalance between endothelial cell tube formation and the parallel development of pericytes [48]. Although the maturation status of vessels within the CIA synovium needs further investigation, vessel immaturity as well as an increased endothelial cell turnover could be an important factor contributing to the hypoxic environment found in the arthritic synovium of mice [19] and humans [6,49], thereby aggravating synovial inflammation. Thus, despite apparent high vascularity, the arthritic synovium remains a rather inhospitable environment, with marked hypoxia and acidosis [6,50], suggesting that blood flow is impaired and insufficient to meet the metabolic demand of inflamed synovial tissue. It might therefore be speculated that just like in cancer, vessel normalisation as a therapeutic intervention could improve the efficacy of immunotherapy in RA.

Using IVM, we detected a significant increase in the diameter of synovial capillaries in arthritic knee synovium. Vessel dilation probably indicates ongoing angiogenesis, since it is one of the first steps preceding vessel sprouting. Capillaries might be in a plastic state, responsive to pro-angiogenic stimuli (for example, VEGF), resulting in increased capillary diameter, remodelling and sprouting of new capillaries. This finding might also suggest that during CIA, the synovium tries to adapt to hypoxia by both angiogenesis and dilation of microvessels. However, increased permeability of capillaries allows the escape of plasma and plasma proteins in addition to the accumulation of leukocytes in the synovium, exacerbating joint inflammation.

The requirement of several angiogenic growth factors for synovial angiogenesis and inflammation during CIA has been shown previously, for example, by neutralising VEGF function [21-23], depletion of midkine [37], or by blocking angiopoietin signalling [33]. Our gene expression results suggest that VEGF-mediated angiogenesis and inflammation during CIA is largely modulated through increased expression of VEGF receptors and co-receptors. NRP-1 not only modulates the function of VEGF during angiogenesis by enhancing the binding of VEGF165 isoform to VEGF-R2 [51], but also interacts directly with VEGF121 [34] and other heparin-binding growth factors, such as HGF, VEGF/B or PLGF [52]. In the rheumatoid synovium, NRP-1 expression can be found on synoviocytes, infiltrating leukocytes and on the endothelium [17,53] and concomitant expression of VEGF165, KDR, and NRP-1 is associated with a high vascular density and increased inflammation [17]. These observations, together with our findings showing increased *Nrp-1 *expression during CIA, suggested that blocking NRP-1 function may affect disease progression by directly reducing synovial angiogenesis and leukocyte infiltration. We applied a monoclonal antibody that targets the VEGF binding site on NRP-1 without interfering with its semaphorin signalling, and which has been shown to reduce neovascularisation in tumour models [28]. Our study revealed that anti-NRP1^B ^is able to inhibit endothelial cell migration induced by arthritic paw extracts, confirming that arthritic tissue contains factors that signal through NRP-1. Treatment with anti-NRP1^B ^significantly attenuated the progression of CIA as assessed by paw swelling and clinical score. Importantly, histological examination of arthritic hind paws demonstrated that this clinical improvement was associated with a reduction in synovial inflammation, pannus formation, as well as destruction of cartilage and bone, supporting the concept of a key role for this ligand-receptor interaction in the pathogenesis of arthritis. The role of NRP-1 in experimental arthritis has been studied before [54]. The prophylactic administration of an anti-NRP-1 peptide diminished disease severity in mouse CIA. Although prophylactic studies offer valuable insight into the molecular mechanisms underlying a disease, care must be taken in translating such results into potential therapeutic application. In contrast, our study employed a therapeutic approach, in which only mice with clinically evident arthritis received anti-NRP1^B^. Our data indicate that NRP-1 signalling is required for the maintenance and progression of established inflammatory arthritis. However, further investigations are necessary to elucidate the exact mechanism by which anti-NRP1^B ^therapy improves inflammatory arthritis.

## Conclusions

While a number of gene expression studies in CIA have been described, the present study was the first to focus on genes associated with angiogenesis, which is a key feature of RA. Our data are compatible with existing theories of angiogenesis in RA, and suggest that synovial angiogenesis results in the formation of dysfunctional vessels primarily caused by an imbalance of pro- and anti-angiogenic factors. Our results confirm NRP-1 as a key player in the pathogenesis of CIA, and support the VEGF/VEGF receptor pathway as a potential therapeutic target in RA.

## Abbreviations

ANG: angiopoietin; ANOVA: analysis of variance; anti-NRP1^B^: phage-derived monoclonal antibody against neuropilin-1; ARP: acidic ribosomal phosphoprotein P0; CII: type II collagen ; CIA: collagen-induced arthritis; CFA: complete Freund's adjuvant; Ct: cycle threshold; FCD: functional capillary density; FGF: fibroblast growth factor; FITC: fluorescein isothiocyanate; FLK: **fetal liver kinase; **FLT: **fms*-*like tyrosine kinase; **GAPDH: glyceraldehyde-3-phosphate dehydrogenase; GUSB: β-glucuronidase; **HGF: hepatocyte growth factor; **HIF-1α: hypoxia inducible factor 1alpha; HMEC: human microvascular endothelial cells; HPRT-1: hypoxanthine guanine phosphoribosyl transferase 1; HSP-1: heat shock protein-1β; IFA: incomplete Freund's adjuvant; IL: interleukin; IGF: insulin-like growth factor; IVM: intravital microscopy; KDR: kinase insert domain receptor; MMP: matrix metalloproteinase; MMLV: Moloney murine leukaemia virus; NRP: neuropilin; PBS: phosphate buffered saline; PLGF: **placental growth factor; **RA: rheumatoid arthritis; SEM: standard error of the mean; TIE: **tyrosine kinase with immunoglobulin-like and EGF-like domains; **TIMP: **tissue inhibitor of metalloproteinases; **TNF: tumour necrosis factor; TSP: thrombospondin; VEGF: vascular endothelial growth factor.

## Competing interests

The authors declare that they have no competing interests.

## Authors' contributions

YR designed and performed all the experiments, data analysis and drafting of the manuscript. EMP, SI and MF assisted in the study design and coordination and oversaw the data analysis and drafting of the manuscript. All authors read and approved the final manuscript for publication.

## References

[B1] Feldmann M Brennan FM Maini RN Rheumatoid arthritis Cell 1996 85 307 310 10.1016/S0092-8674(00)81109-5 8616886

[B2] Paleolog EM The vasculature in rheumatoid arthritis: cause or consequence? Int J Exp Pathol 2009 90 249 261 10.1111/j.1365-2613.2009.00640.x 19563609 PMC2697549

[B3] Khong T Larsen H Raatz Y Paleolog E Angiogenesis as a therapeutic target in arthritis: learning the lessons of the colorectal cancer experience Angiogenesis 2007 10 243 258 10.1007/s10456-007-9081-1 17805984

[B4] Rooney M Condell D Quinlan W Daly L Whelan A Feighery C Bresnihan B Analysis of the histologic variation of synovitis in rheumatoid arthritis Arthritis Rheum 1988 31 956 963 10.1002/art.1780310803 2457377

[B5] Walsh D Wade M Mapp P Blake D Focally Regulated Endothelial Proliferation and Cell Death in Human Synovium American Journal of Pathology 1998 152 691 702 9502411 PMC1858385

[B6] Lund-Olesen K Oxygen tension in synovial fluids Arthritis Rheum 1970 13 769 776 10.1002/art.1780130606 5495389

[B7] Semble EL Turner RA McCrickard EL Rheumatoid arthritis and osteoarthritis synovial fluid effects on primary human endothelial cell cultures J Rheumatol 1985 12 237 241 3897531

[B8] Koch AE Harlow LA Haines GK Amento EP Unemori EN Wong WL Pope RM Ferrara N Vascular endothelial growth factor. A cytokine modulating endothelial function in rheumatoid arthritis J Immunol 1994 152 4149 4156 7511670

[B9] Paleolog EM Young S Stark AC McCloskey RV Feldmann M Maini RN Modulation of angiogenic vascular endothelial growth factor by tumor necrosis factor alpha and interleukin-1 in rheumatoid arthritis Arthritis Rheum 1998 41 1258 1265 10.1002/1529-0131(199807)41:7<1258::AID-ART17>3.0.CO;2-1 9663484

[B10] Scott BB Zaratin PF Colombo A Hansbury MJ Winkler JD Jackson JR Constitutive expression of angiopoietin-1 and -2 and modulation of their expression by inflammatory cytokines in rheumatoid arthritis synovial fibroblasts J Rheumatol 2002 29 230 239 11838839

[B11] Fava RA Olsen NJ Spencer-Green G Yeo TK Berse B Jackman RW Senger DR Dvorak HF Brown LF Vascular permeability factor/endothelial growth factor (VPF/VEGF): accumulation and expression in human synovial fluids and rheumatoid synovial tissue The Journal of Experimental Medicine 1994 180 341 346 10.1084/jem.180.1.341 8006592 PMC2191547

[B12] Shahrara S Volin MV Connors MA Haines GK Koch AE Differential expression of the angiogenic Tie receptor family in arthritic and normal synovial tissue Arthritis Research 2002 4 201 208 10.1186/ar407 12010571 PMC111023

[B13] Koch AE Halloran MM Hosaka S Shah MR Haskell CJ Baker SK Panos RJ Haines GK Bennett GL Pope RM Ferrara N Hepatocyte growth factor. A cytokine mediating endothelial migration in inflammatory arthritis Arthritis Rheum 1996 39 1566 1575 10.1002/art.1780390917 8814069

[B14] Hosaka S Shah MR Barquin N Haines GK Koch AE Expression of basic fibroblast growth factor and angiogenin in arthritis Pathobiology 1995 63 249 256 10.1159/000163957 8724206

[B15] Sone H Sakauchi M Takahashi A Suzuki H Inoue N Iida K Shimano H Toyoshima H Kawakami Y Okuda Y Matsuo K Yamada N Elevated levels of vascular endothelial growth factor in the sera of patients with rheumatoid arthritis correlation with disease activity Life Sci 2001 69 1861 1869 10.1016/S0024-3205(01)01264-4 11693266

[B16] Ballara S Taylor PC Reusch P Marme D Feldmann M Maini RN Paleolog EM Raised serum vascular endothelial growth factor levels are associated with destructive change in inflammatory arthritis Arthritis & Rheumatism 2001 44 2055 2064 10.1002/1529-0131(200109)44:9<2055::AID-ART355>3.0.CO;2-2 11592367

[B17] Ikeda M Hosoda Y Hirose S Okada Y Ikeda E Expression of vascular endothelial growth factor isoforms and their receptors Flt-1, KDR, and neuropilin-1 in synovial tissues of rheumatoid arthritis J Pathol 2000 191 426 433 10.1002/1096-9896(2000)9999:9999<::AID-PATH649>3.0.CO;2-E 10918218

[B18] Williams RO Feldmann M Maini RN Anti-tumor necrosis factor ameliorates joint disease in murine collagen-induced arthritis Proc Natl Acad Sci USA 1992 89 9784 9788 10.1073/pnas.89.20.9784 1409699 PMC50217

[B19] Etherington PJ Winlove P Taylor P Paleolog E Miotla JM VEGF release is associated with reduced oxygen tensions in experimental inflammatory arthritis Clin Exp Rheumatol 2002 20 799 805 12508771

[B20] DeBusk LM Chen Y Nishishita T Chen J Thomas JW Lin PC Tie2 receptor tyrosine kinase, a major mediator of tumor necrosis factor alpha-induced angiogenesis in rheumatoid arthritis Arthritis Rheum 2003 48 2461 2471 10.1002/art.11213 13130465

[B21] Lu J Kasama T Kobayashi K Yoda Y Shiozawa F Hanyuda M Negishi M Ide H Adachi M Vascular endothelial growth factor expression and regulation of murine collagen-induced arthritis The Journal of Immunology 2000 164 5922 5927 10820274 10.4049/jimmunol.164.11.5922

[B22] Sone H Kawakami Y Sakauchi M Nakamura Y Takahashi A Shimano H Okuda Y Segawa T Suzuki H Yamada N Neutralisation of vascular endothelial growth factor prevents collagen-induced arthritis and ameliorates established disease in mice Biochemical and Biophysical Research Communications 2001 281 562 568 10.1006/bbrc.2001.4395 11181084

[B23] Miotla J Maciewicz R Kendrew J Feldmann M Paleolog E Treatment with soluble VEGF receptor reduces disease severity in murine collagen-induced arthritis Laboratory Investigation 2002 80 1195 1205 10950110 10.1038/labinvest.3780127

[B24] Clavel G Valvason C Yamaoka K Lemeiter D Laroche L Boissier MC Bessis N Relationship between angiogenesis and inflammation in experimental arthritis Eur Cytokine Netw 2006 17 202 210 17194641

[B25] Ibrahim SM Koczan D Thiesen HJ Gene-expression profile of collagen-induced arthritis J Autoimmun 2002 18 159 167 10.1006/jaut.2001.0580 11908948

[B26] Gierer P Ibrahim S Mittlmeier T Koczan D Moeller S Landes J Gradl G Vollmar B Gene expression profile and synovial microcirculation at early stages of collagen-induced arthritis Arthritis Res Ther 2005 7 R868 876 10.1186/ar1754 15987489 PMC1175036

[B27] Miller EJ Structural studies on cartilage collagen employing limited cleavage and solubilization with pepsin Biochemistry 1972 11 4903 4909 10.1021/bi00776a005 4565026

[B28] Pan Q Chanthery Y Liang WC Stawicki S Mak J Rathore N Tong RK Kowalski J Yee SF Pacheco G Ross S Cheng Z Le Couter J Plowman G Peale F Koch AW Wu Y Bagri A Tessier-Lavigne M Watts RJ Blocking neuropilin-1 function has an additive effect with anti-VEGF to inhibit tumor growth Cancer Cell 2007 11 53 67 10.1016/j.ccr.2006.10.018 17222790

[B29] Pfaffl MW A new mathematical model for relative quantification in real-time RT-PCR Nucleic Acids Res 2001 29 e45 10.1093/nar/29.9.e45 11328886 PMC55695

[B30] Liem HH Cardenas F Tavassoli M Poh-Fitzpatrick MB Muller-Eberhard U Quantitative determination of hemoglobin and cytochemical staining for peroxidase using 3,3´5,5´tetramethylbenzidine dihydrochloride, a safe substitute for benzidine Analytical Biochemistry 1976 98 388 393 91331 10.1016/0003-2697(79)90157-x

[B31] Veihelmann A Szczesny G Nolte D Krombach F Refior HJ Messmer K A novel model for the study of synovial microcirculation in the mouse knee joint in vivo Res Exp Med (Berl) 1998 198 43 54 10.1007/s004330050088 9706669

[B32] Stephens RW Ghosh P Taylor TK Gale CA Swann JC Robinson RG Webb J The origins and relative distribution of polysaccharides in rheumatoid and osteoarthritic fluids Journal of Rheumatology 1975 2 393 300 1206671

[B33] Malik NM Jin P Raatz Y Sumariwalla PF Kiriakidis S Shepard M Feldmann M Paleolog EM Regulation of the angiopoietin-Tie ligand-receptor system with a novel splice variant of Tie1 reduces the severity of murine arthritis Rheumatology (Oxford) 49 1828 1839 20547659 10.1093/rheumatology/keq163

[B34] Pan Q Chathery Y Wu Y Rathore N Tong RK Peale F Bagri A Tessier-Lavigne M Koch AW Watts RJ Neuropilin-1 binds to VEGF121 and regulates endothelial cell migration and sprouting J Biol Chem 2007 282 24049 24056 10.1074/jbc.M703554200 17575273

[B35] Muz B Khan MN Kiriakidis S Paleolog EM Hypoxia. The role of hypoxia and HIF-dependent signalling events in rheumatoid arthritis Arthritis Res Ther 2009 11 201 10.1186/ar2568 19222864 PMC2688222

[B36] Rundhaug JE Matrix metalloproteinases and angiogenesis J Cell Mol Med 2005 9 267 285 10.1111/j.1582-4934.2005.tb00355.x 15963249 PMC6740080

[B37] Maruyama K Muramatsu H Ishiguro N Muramatsu T Midkine, a heparin-binding growth factor, is fundamentally involved in the pathogenesis of rheumatoid arthritis Arthritis Rheum 2004 50 1420 1429 10.1002/art.20175 15146411

[B38] Takada T Toriyama K Muramatsu H Song XJ Torii S Muramatsu T Midkine, a retinoic acid-inducible heparin-binding cytokine in inflammatory responses: chemotactic activity to neutrophils and association with inflammatory synovitis J Biochem 1997 122 453 458 10.1093/oxfordjournals.jbchem.a021773 9378726

[B39] Yukioka K Inaba M Furumitsu Y Yukioka M Nishino T Goto H Nishizawa Y Morii H Levels of hepatocyte growth factor in synovial fluid and serum of patients with rheumatoid arthritis and release of hepatocyte growth factor by rheumatoid synovial fluid cells J Rheumatol 1994 21 2184 2189 7699616

[B40] Kusada J Otsuka T Matsui N Hirano T Asai K Kato T Immuno-reactive human epidermal growth factor (h-EGF) in rheumatoid synovial fluids Nihon Seikeigeka Gakkai Zasshi 1993 67 859 865 8409646

[B41] Booth G Newham P Barlow R Raines S Zheng B Han S Gene expression profiles at different stages of collagen-induced arthritis Autoimmunity 2008 41 512 521 10.1080/08916930802095210 18608173

[B42] La Cava A Alviggi C Matarese G Unraveling the multiple roles of leptin in inflammation and autoimmunity J Mol Med 2004 82 4 11 10.1007/s00109-003-0492-1 14556053

[B43] Salvi R Peclat V So A Busso N Enhanced expression of genes involved in coagulation and fibrinolysis in murine arthritis Arthritis Res 2000 2 504 512 10.1186/ar132 11056680 PMC17822

[B44] Afuwape AO Kiriakidis S Paleolog EM The role of the angiogenic molecule VEGF in the pathogenesis of rheumatoid arthritis Histol Histopathol 2002 17 961 972 12168808 10.14670/HH-17.961

[B45] Fiedler U Augustin HG Angiopoietins: a link between angiogenesis and inflammation Trends Immunol 2006 27 552 558 10.1016/j.it.2006.10.004 17045842

[B46] Kennedy A Ng CT Biniecka M Saber T Taylor C O'Sullivan J Veale DJ Fearon U Angiogenesis and blood vessel stability in inflammatory arthritis Arthritis Rheum 62 711 721 20187131 10.1002/art.27287

[B47] Izquierdo E Canete JD Celis R Santiago B Usategui A Sanmarti R Del Rey MJ Pablos JL Immature blood vessels in rheumatoid synovium are selectively depleted in response to anti-TNF therapy PLoS ONE 2009 4 e8131 10.1371/journal.pone.0008131 19956574 PMC2779850

[B48] Greenberg JI Shields DJ Barillas SG Acevedo LM Murphy E Huang J Scheppke L Stockmann C Johnson RS Angle N Cheresh DA A role for VEGF as a negative regulator of pericyte function and vessel maturation Nature 2008 456 809 813 10.1038/nature07424 18997771 PMC2605188

[B49] Sivakumar B Akhavani MA Winlove CP Taylor PC Paleolog EM Kang N Synovial hypoxia as a cause of tendon rupture in rheumatoid arthritis J Hand Surg [Am] 2008 33 49 58 10.1016/j.jhsa.2007.09.002 18261665

[B50] Levick JR Hypoxia and acidosis in chronic inflammatory arthritis; relation to vascular supply and dynamic effusion pressure J Rheumatol 1990 17 579 582 2359066

[B51] Soker S Takashima S Miao HQ Neufeld G Klagsbrun M Neuropilin-1 is expressed by endothelial and tumor cells as an isoform-specific receptor for vascular endothelial growth factor Cell 1998 92 735 745 10.1016/S0092-8674(00)81402-6 9529250

[B52] Sulpice E Plouet J Berge M Allanic D Tobelem G Merkulova-Rainon T Neuropilin-1 and neuropilin-2 act as coreceptors, potentiating proangiogenic activity Blood 2008 111 2036 2045 10.1182/blood-2007-04-084269 18065694

[B53] Kim WU Kang SS Yoo SA Hong KH Bae DG Lee MS Hong SW Chae CB Cho CS Interaction of vascular endothelial growth factor 165 with neuropilin-1 protects rheumatoid synoviocytes from apoptotic death by regulating Bcl-2 expression and Bax translocation J Immunol 2006 177 5727 5735 17015762 10.4049/jimmunol.177.8.5727

[B54] Kong JS Yoo SA Kim JW Yang SP Chae CB Tarallo V De Falco S Ryu SH Cho CS Kim WU Anti-neuropilin-1 peptide inhibition of synoviocyte survival, angiogenesis, and experimental arthritis Arthritis Rheum 62 179 190 20039409 10.1002/art.27243

[B55] Whitehead Institute for Biomedical Research http://www.genome.wi.mit.edu

